# Wild and cultivated olive tree genetic diversity in Greece: a diverse resource in danger of erosion

**DOI:** 10.3389/fgene.2023.1298565

**Published:** 2023-12-04

**Authors:** Nikolaos Tourvas, Ioannis Ganopoulos, Georgios Koubouris, George Kostelenos, Ioannis Manthos, Christos Bazakos, Vasileios Stournaras, Athanassios Molassiotis, Filippos Aravanopoulos

**Affiliations:** ^1^ Laboratory of Forest Genetics, Faculty of Agriculture, Forestry and Natural Environment, Aristotle University of Thessaloniki, Thessaloniki, Greece; ^2^ Institute of Plant Breeding and Genetic Resources, Hellenic Agricultural Organization (ELGO) DIMITRA, Thessaloniki-Thermi, Greece; ^3^ Institute of Olive Tree, Subtropical Crops and Viticulture, Hellenic Agricultural Organization (ELGO) DIMITRA, Chania, Greece; ^4^ Kostelenos Olive Nurseries, Poros-Trizinias, Greece; ^5^ Department of Nut Trees, Institute of Plant Breeding and Genetic Resources, Hellenic Agricultural Organization (ELGO) DIMITRA, Neo Krikello-Lamia, Greece; ^6^ Department of Comparative Development and Genetics, Max Planck Institute for Plant Breeding Research, Cologne, Germany; ^7^ Department of Olive and Horticultural Crops, Institute of Olive Tree, Subtropical Crops and Viticulture, Hellenic Agricultural Organization (ELGO) DIMITRA, Kalamata, Greece; ^8^ Laboratory of Pomology, Department of Horticulture, Aristotle University of Thessaloniki, Thessaloniki-Thermi, Greece

**Keywords:** crop evolution, cultivars, genetic diversity, genetic pollution, gene flow, microsatellites, Olea europaea, wild crop relatives

## Abstract

The genetic relationships between Greek wild olive tree populations and cultivars were investigated. A total of 219 wild genotypes and 67 cultivar genotypes were analyzed by employing 10 SSR markers. Data evidenced that the wild populations exhibited high levels of genetic diversity and exclusively host 40% of the total number of alleles detected. Inbreeding was observed within populations, probably as a consequence of their fragmented spatial distribution. The genetic differentiation between cultivars and wild individuals, as well as within wild populations, was low. Nevertheless, three gene pools of wild trees were detected, corresponding to the geographical areas of Northeastern Greece, Peloponnese-Crete and Epirus. Most cultivars clustered in a separate group, while the rest of them formed a heterogenous group with membership coefficients akin to the three wild olive clusters. Regarding the history of olive cultivation in Greece, bidirectional gene flow was detected between populations of Peloponnese-Crete and the gene pool that composes some of Greece’s most important cultivars, such as "Koroneiki” and “Mastoidis”, which is inferred as an indication of a minor domestication event in the area. A strategy for the protection of Greek-oriented olive genetic resources is proposed, along with suggestions for the utilization of the genetically diverse wild resources with regard to the introgression of traits of agronomical interest to cultivars.

## 1 Introduction

The olive tree (*Olea europaea* subsp. *europaea*) is recognised as an emblematic plant species of the Mediterranean basin. It is found in two different types, known since antiquity as reported by Theophrastus (1916), wild (*O. europaea* subsp. *europaea* var. *sylvestris*) and cultivated (*O. europaea* subsp. *europaea* var. *europaea*) (Green, 2002). Βoth forms (wild and cultivated) are interfertile (Besnard et al., 2000) with 23 pairs (2n = 2x = 46) of chromosomes (Contento et al., 2002) and excellent grafting compatibility (Mulas and Deidda, 1998). The reproduction of wild forms takes place sexually, assisted by wind, as well as birds that contribute to seed dispersion (Alcántara and Rey, 2003). The presence-absence of wild olive trees is an excellent biomarker of the Mediterranean Floristic Region (Rubio et al., 2002). A plethora of cultivated accessions exist and are conserved in banks worldwide (Bartolini and Cerreti, 2008) and at the country level (Barranco and Rallo, 2000; Khadari et al., 2003; Gemas et al., 2004; Bracci et al., 2009; Haouane et al., 2011; Yoruk and Taskin, 2014; Koubouris et al., 2019). Based on the consensus from archeological, paleobotanical and genetic data, these cultivars originate almost entirely from ancestral populations of Mediterranean wild olive trees, also known as oleasters (Kaniewski et al., 2012; Besnard et al., 2018; Julca et al., 2023). Nowadays, oleasters are located in humid and subhumid thermo-Mediterranean areas where no frost is observed (Martínez and Gandullo, 1987; Carrión et al., 2010).

It is known from multiple studies that wild olive trees of the Mediterranean region are genetically distinguished into the Western (WW) and Eastern Mediterranean (WE) groups (Breton et al., 2006; Belaj et al., 2007; Diez et al., 2015). The diversity of cultivated genotypes is better described by the formation of three groups - Western (Q1), Central (Balkan and Italian peninsula - Q2), and Eastern Mediterranean (Q3) (Haouane et al., 2011; Diez et al., 2015; Besnard et al., 2018). Additionally, Q2 exhibits the highest chloroplast diversity compared to Q1 and Q3 (Diez et al., 2015), with the Aegean region as a hotspot. Two hypotheses have been proposed regarding the origin of the cultivated olive tree. The first describes a unique domestication event that occurred in the Eastern Mediterranean group (Q3) (Besnard et al., 2013a; 2013b; Khadari and El Bakkali, 2018). The main evidence in support of this hypothesis is the fact that 90% of the cultivated genotypes carry the Eastern Mediterranean chloroplast haplotype E1.1 (Besnard et al., 2013b). Nuclear DNA markers have confirmed the close genetic relationship of modern cultivars with the Eastern Mediterranean wild olive gene pool (Lumaret et al., 2004; Breton et al., 2006). However, other studies based on the results of nuclear microsatellite marker studies support the existence of two or more domestication events in the eastern (Q3) and central Mediterranean (Q2) groups (Breton et al., 2009; Diez et al., 2015). High-throughput sequencing analyzes (Gros-Balthazard et al., 2019; Julca et al., 2020) tend to support the existence of one major domestication event, but point to the existence of additional events of secondary magnitude. In contrast, Jiménez-Ruiz et al. (2020) analysed the transposable elements of cultivars and wild individuals and supported the presence of two independent domestication events. Recently, a more in-depth analysis of the olive genome, including copy number variations, was performed by Bazakos et al. (2023) and uncovered several candidate genes involved in selective sweeps. Furthermore, a GWAS analysis for morphological and agronomic traits allowed the detection of significant associations to support the next steps of olive tree breeding (Bazakos et al., 2023). For wild olives, Unver et al. (2017) has provided the only to date available reference genome, while Zunino et al. (2023) employed a targeted sequencing approach to characterize 400 trees, mainly from the Western Mediterranean basin. They uncovered high genetic differentiation between some wild populations and cultivars, which implied the presence of genuine wild olives. Nevertheless, they highlighted several previously thought genuine wild populations as admixed, even within remote sites away from oleiculture activity. In another recent work, Mariotti et al. (2020) employed chloroplast and nuclear markers to analyse multiple genera of cultivated, ancient and wild genotypes among *O. europaea* and the Oleaceae family. Using population and comparative genomics they hinted of upcoming opportunities for uncovering the origin of agronomic and ecological traits of importance.

In order to prevent a possible genetic erosion of the olive species, research, evaluation and conservation of its genetic resources are of the utmost importance. A thorough analysis of the genetic wealth of olive genotypes will lead to new approaches to deciphering olive domestication and shed light on the distribution of genetic diversity (Baldoni et al., 2006). Furthermore, the estimation and comparison of the genetic diversity of wild and cultivated olives targeted at specific areas is the key to a better understanding of the reduction in genetic diversity resulting from the domestication and intensive use of agricultural practices.

The present study examines the level of genetic diversity in wild olive trees and its distribution in the north-east Mediterranean basin, an area of intense olive cultivation where wild and cultivated forms have long coexisted (Terral et al., 2004). In addition, cultivar samples from reference collections and from the field were uniquely genotyped to: 1) perform a comparative analysis of the genetic diversity of wild and cultivated olive trees; 2) shed light on genetic structure patterns within wild populations and 3) between wild and cultivated trees, and 4) investigate the occurrence of gene flow between these gene pools.

## 2 Materials and methods

### 2.1 Sampling and DNA extraction

**FIGURE 1:**
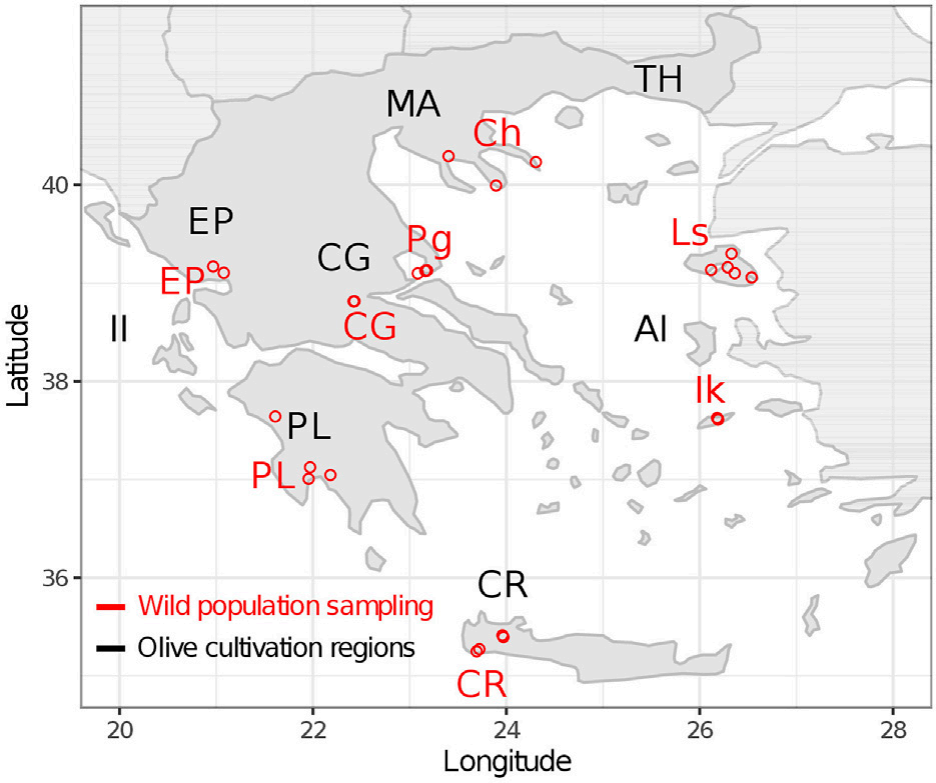
Wild olive tree sampling. Sampled populations are indicated with red color. Geographical regions of Greece where olive cultivars originate from are indicated with black color.

A total of 219 wild olive tree samples were collected from eight geographical regions in Greece (Table 1). In every region, multiple sampling plots were utilized as extended populations were unavailable (Figure 1). We also hypothesized that spreading our sampling effort would increase the chances of collecting samples with low genetic affinity to cultivars. The sample distance was at a minimum of 20 m. Young leaves were collected from individuals with the oleaster phenotype (trees or shrubs, often multi-stemmed with spinescent branches). Where wild populations were unavailable, root suckers from centennial cultivar trees were sampled. For the Chalkidiki population, two oleaster samples were also received from the herbarium of the School of Forestry and Natural Environment, Thessaloniki Aristotle University - TAUF. These were collected in the area of Agion Oros in 1999. Coordinates for the sampling plots of each population are provided in Supplementary Table S1.

**TABLE 1:** Wild and cultivated olive tree sample origin.

Gene pool	Sample Origin	Sample Origin abbreviation	Geographic region	Geographic region abbreviation	Number of samples
Wild olive trees (W)	Crete	CR	Crete	CR	30
	Chalkidiki	Ch	Macedonia	MA	33
	Lesvos	Ls	Aegean Islands	AI	30
	Peloponnese	PL	Peloponnese	PL	29
	Epirus	EP	Epirus	EP	30
	Central Greece	CG	Central Greece	CG	30
	Ikaria	Ik	Aegean Islands	AI	30
	Pagasitikos Bay	Pg	Aegean Islands	AI	7
Cultivars (C)	NOGB Reference samples	C	^a^	^a^	46
	Greek cultivars (Field collection)		^a^	^a^	14
	Monumental tree “Throuba Naxou” and commercial clone “Throuba”		Aegean Islands	AI	2
	Spanish cultivars		Spain	ES	2
Outgroup (OUT)	*Olea europaea* subsp. *cuspidata*	OUT	Asia/Africa	OUT	3
Total					286

^a^ see Table 2.

Forty six reference Greek cultivar leaf samples were provided by the National Olive Germplasm Bank of Greece (NOGB), Institute of Olive Tree, Subtropical Crops and Viticulture (IOSV), Hellenic Agricultural Organization “ELGO - Dimitra” (Chania, Southern Greece). This collection was enriched by 14 additional samples from olive groves nearby wild samples, one commercially available clone “Throuba”, two Spanish cultivars (“Arbequina”, “Picual”), a monumental tree from Naxos island with an estimated age of over 3,000 years (Bazakos et al., 2023) and three *O. europaea* subsp*. Cuspidata* accessions as outgroups (Table 1, Table 2). Plant tissue was stored at −80°C until DNA extraction with the DNeasy Plant Pro Kit (Qiagen Inc., Valencia, CA).

**TABLE 2:** Principal area of cultivation of olive cultivars investigated in the present study.

Cultivar	Principal area of cultivation	Region	Region abbreviation
ADRAMYTINI	Lesvos island, Andros island	Aegean Islands	AI
AGOUROMANAKOLIA	Kynouria, Argolida, Korinthia	Peloponnese	PL
AMYGDALOLIA	Attika, Evia, Voeotia	Central Greece	CG
ASPROLIA ALEXANDROUPOLIS	Alexandroupoli	Thrace	TH
ASPROLIA LEFKADOS	Lefkada and Meganissi islands, Aetoloakarnania	Ionian Islands - Western Central Greece	II - CG
CHALKIDIKIS	Chalkidiki	Macedonia	MA
CHONDROLIA CHALKIDIKIS	Chalkidiki	Macedonia	MA
CHRYSOLIA	Outgroup	Outgroup	OUT
DAFNELIA	Islands of Chios, Samos, Ikaria, Andros, Siros, Naxos, Sifnos (all Central and Eastern Aegean islands)	Aegean Islands	AI
FRANTOIO RODOU	Rhodos island and Italy (Italian origin)	Aegean Islands	AI
GAIDOURELIA	Kynouria	Peloponnese	PL
GALATISTAS AG.OROUS	Agion Oros and Monopigado	Macedonia	MA
KALAMON	Lakonia, Messinia, Ilia, Aetoloakarnania, Fthiotioda Kynoyria	Peloponnese	PL
KALOKERIDA	Kerkira island	Ionian Islands	II
KAROLIA	Lesvos island	Aegean Islands	AI
KARYDOLIA	Voeotia, Fokida	Macedonia	CG
KOLYBADA	Attika and Tinos island	Aegean Islands	AI-CG
KOLYREIKI	Ilia	Peloponnese	PL
KONSERVOLIA/AMFISSIS	Igoumenitsa, Magnissia, Evia, Fthiotida, Aetoloakarnania, Fokida, Achaia	(Western) Central Greece - Ionian Islands	CG-II
KORONEIKI	Crete, West and South Peloponnese, Central and South Aegean and Ionian islands	Crete - Peloponnese	CR-PL
KOTHREIKI	Argolida, Fokida, Veoetia, Korinthia, Fthiotida	Central Greece	CG-PL
KOUTSOURELIA	Korinthia, Lakonia and Aetoloakarnania	Central Greece - Peloponnese	CG-PL
LEFKOLIA SERRON	Serres	Macedonia	MA
LIANOLIA KERKYRAS	Kerkyra and Paxi island, Igoumenitsa and Preveza	Ionian Islands	II
LIANOMANAKO TYROY	Kynouria	Peloponnese	PL
MAKRIS	Evros, Rodopi and Samothraki island	Thrace	TH
MASTOIDIS/TSOUNATI	Crete, Lakonia, Messinia, Arkadia, Ilia and Achaia	Crete - Peloponnese	CR-PL
MATOLIA ILIAS	Ilia	Peloponnese	PL
MAVRELIA	Messinia	Peloponnese	PL
MAVRELIA SERRON	Serres	Macedonia	MA
MAVROLIA LEFKADOS	Lefkada island	Ionian Islands	II
MEGARΕITIKI	Attika, Argolida, Veoetia, Fthiotida, Fokida, Kynouria, Achaia	Central Greece - Peloponnese	CG-PL
MYRTOLIA	Lakonia	Peloponnese	PL
NTOPIA ZAKYNTHOU	Zakynthos island	Ionian Islands	II
PETROLIA	Serres	Macedonia	MA
PIERIAS	Pieria	Macedonia	MA
PIERIAS (SKOTINIS)	Pieria	Macedonia	MA
PIKROLIA	Kerkira island	Ionian Islands	II
RACHATI	Achaia and Aetoloakarnania	Peloponnese - Central Greece	CG-PL
STROGGYLOLIA	Chalkidiki	Macedonia	MA
THIAKI	Kefalonia and Ithaki islands	Ionian Islands	II
THROUBA THASOU	Thassos island, Kavala	Aegean Islands	AI-MA
THROUBOLIA	Crete (Rethymno, Herakleion), Evia and many Aegean islands	Aegean Islands - Crete	AI-CR
TRAGOLIA	Messinia, Lakonia	Peloponnese	PL
VALANOLIA	Lesvos, Chios and Skyros islands	Aegean Islands	AI
VASILIKADA	Kerkira island, North Evia	Central Greece	CG
Ls-C-N-01	Lesvos	Aegean Islands	AI
Ls-C-N-02	Lesvos	Aegean Islands	AI
Ls-C-N-03	Lesvos	Aegean Islands	AI
Ls-C-N-04	Lesvos	Aegean Islands	AI
Ls-C-N-05	Lesvos	Aegean Islands	AI
Ls-C-N-06	Lesvos	Aegean Islands	AI
Ls-C-N-07	Lesvos	Aegean Islands	AI
Ik-C-N-01	Ikaria	Aegean Islands	AI
Ik-C-N-02	Ikaria	Aegean Islands	AI
Ik-C-N-03	Ikaria	Aegean Islands	AI
Ik-C-N-04	Ikaria	Aegean Islands	AI
CR-C-N-01	Crete	Crete	CR
CR-C-N-02	Crete	Crete	CR
CR-C-N-03	Crete	Crete	CR
THROUBA	Crete (Rethymno, Herakleion), Evia and many Aegean islands	Aegean Islands	ΑΙ
THROUBA_NAXOU	Naxos island	Aegean Islands	ΑΙ
ARBEQUINA	Spain	Spain	ES
PICUAL	Spain	Spain	ES
OUT-1	-	Outgroup	OUT
OUT-3	-	Outgroup	OUT
OUT-4	-	Outgroup	OUT

### 2.2 Microsatellite genotyping

A set of 11 microsatellite markers (Sefc et al., 2000; Carriero et al., 2002; Cipriani et al., 2002; Díaz et al., 2006) was selected for genotyping using i) reproducibility and ii) ease of scoring as criteria (Doveri et al., 2008; Baldoni et al., 2009). A touchdown PCR procedure was used: 95°C for 5 min, 10 touchdown cycles: 95°C for 20 s, 60°C for combination A and 65°C for combination B for 30 s (−1°C step/cycle) and 72°C for 30 s. It was followed by 25 cycles: 95°C for 20 s, 50°C for combination A and 54°C for combination B for 30 s, 72°C for 30 s, final extension step at 72°C for 10 min. Fragment analysis was conducted in an ABI 3730xl (Applied Biosystems, Foster City, CA, United States) with GeneScan 500 LIZ size standard and results were recorded with GeneMapper v4. Cultivar reference samples were genotyped twice to avoid sample mix-up (two independent DNA extractions and PCR amplifications). PCR amplifications were repeated for a random sample of 30 individuals (∼10% of the whole data set). These data were used to calculate i) error rate per reaction and ii) per allele (Hoffman and Amos, 2005).

### 2.3 Data analysis

Statistical analysis was performed in R 4.1.3 (R Core Team, 2021). Basic population statistics were estimated with poppr (Kamvar et al., 2014), hierfstat (Goudet, 2005), adegenet (Jombart, 2008), and genepop (Rousset, 2008). Synonymy-clonality (different samples with identical multilocus genotypes-MLG) was determined in poppr. To avoid cases of samples differing only due to genotyping error and mutations in hyper-variable loci, MLGs were collapsed by dissimilarity distance (denoted in the rest of the text as “contracted MLGs”). The minimum dissimilarity distance at which two individuals would be considered as “contracted MLGs”, was decided by identifying an initial small peak in the histogram of pairwise distances, as suggested by Arnaud-Haond et al. (2007). Due to the limited sample size, the population of Pagasitikos Bay was not evaluated for population level inferences. To preserve custom code produced for this analysis a new R package was developed named *PopGenUtils* (https://github.com/nikostourvas/PopGenUtils). It was used to calculate the probability of identity (PID) (Paetkau and Strobeck, 1994) and the more conservative statistic (PIDsibs) (Taberlet and Luikart, 1999). Polymorphic information content was estimated with polysat (Clark and Jasieniuk, 2011).

$$PID={2\left(\sum p_{i}^{2}\right)}^{2}-\;\sum p_{i}^{4}$$

$$PIDsibs=0.25+\left(0.5\sum p_{i}^{2}\right)+\left[{0.5\left(\sum p_{i}^{2}\right)}^{2}\right]-\left(0.25\sum p_{i}^{4}\right)$$

where *p*_*i*_ is the frequency of allele i.

Rarefaction curves were produced with vegan (Oksanen et al., 2020). Genetic structure was evaluated using G_ST_ as calculated in strataG (Archer et al., 2017). The admixture and the correlated allele frequency models (Falush et al., 2003) of STRUCTURE 2.3.4 software (Pritchard et al., 2000) via the multi-core implementation provided by Structure_threader (Pina-Martins et al., 2017), were used. Wang’s (2017) suggestions to avoid bias due to asymmetrical sampling were implemented. In particular, i) the alternative ancestry prior was used by setting POPALPHAS = 1 and ii) the default ALPHA value 1) was reduced to ALPHA = 0.2 based on the suggestion of 1/K (where K is the expected number of groups in the data set). Burn-in period was set to 5 × 10^5^, followed by 10^6^ iterations for K = 1 up to K = 8 with 20 repeats for each K value. The “distruct for many K’s” pipeline from CLUMPAK (Kopelman et al., 2015) was implemented to aggregate results. Outgroup samples were not used in genetic structure analysis as they represent an extreme case of asymmetric sampling (Neophytou, 2014; Puechmaille, 2016). Membership proportions were visualized with pophelper (Francis, 2017).

AssignPOP 1.2.4 (Chen et al., 2018) was used to perform assignment tests with the SVM prediction algorithm. This software splits the given data set in “training” and “test” data based on cross-validation (in this case 100 iterations). A dimension reduction step follows, using PCA analysis and a machine learning algorithm classifies the samples. The developed predictive model is evaluated using the withheld “test” data which helps avoid high-grading bias (Anderson, 2010).

The presence of one up to five gene flow events between gene pools was investigated with TreeMix (Pickrell and Pritchard, 2012), with gene pools being determined based on Structure results. Ten independent runs were executed for each evolutionary scenario. Data input for TreeMix was prepared by calculating the mean and the standard deviation of the absolute length of each microsatellite. The function genind2treemix is provided in the PopGenUtils package to produce such an input file. Code from the R package OptM (Fitak, 2021) was used to plot mean likelihood values for different scenarios of gene flow events.

The complexity of the analyses offered by modern statistical tools has led to problems of reproducibility of research results (Gilbert et al., 2012). To counter that, all statistical analyses were conducted in a container-based computing environment (https://github.com/nikostourvas/forest_popgen), which included the operating system, software, scripts and raw data following the recommendations of Marwick et al. (2018). This was registered in a Zenodo digital repository (10.5281/zenodo.8104610) and is available to any party interested in regenerating and verifying the results of this work.

## 3 Results

### 3.1 Data quality check and clone detection

Locus GAPU103A displayed suboptimal PCR amplification and was dropped from analyses. Following the PCR repeatability test in 30 individuals, 10 errors were recorded (error rate per reaction: 1.69%). All cases were with regards to erroneous scoring of an allele (error rate per allele: 0.85%) (Table 3). Polymorphic Information Content (PIC) varied from 0.926 for marker DCA09 to 0.735 for marker DCA13. To assess whether the remaining 10 microsatellite loci provided sufficient statistical power to distinguish olive tree samples and elucidate patterns of fine genetic structure, a statistical resampling procedure was executed. PID and PIDsibs values were calculated by sampling with replacement over loci 1,000 times for 1-9 loci (Supplementary Table S2, Supplementary Figure S2A). The same procedure was followed for calculating the number of detected MLGs for different combinations of the genotyped loci (Supplementary Figure S2B). Random combinations of as few as three loci were enough to achieve low PID (median PID = 2.12 × 10^−5^) and discriminate most MLGs. The overall probability of identity (PID) was 1.60 × 10^−16^, while overall PIDsibs was 1.25 × 10^−5^.

**TABLE 3:** Error rate per reaction/allele and Polymorphic information content for the 10 microsatellite loci analyzed.

Locus	Number of reactions	Number of errors per reaction	Number of errors per allele	Error rate per reaction	Error rate per allele	PIC
DCA09	59	3	3	0.05	0.025	0.926
UDO43	59	2	2	0.0333	0.01667	0.913
DCA16	59	1	1	0.0167	0.0083	0.895
DCA18	60	2	2	0.0333	0.0167	0.889
DCA14	60	0	0	0	0	0.878
IAS-oli23	60	0	0	0	0	0.868
DCA03	60	1	1	0.0167	0.0083	0.837
GAPU71B	54	1	1	0.0167	0.0083	0.818
DCA05	60	0	0	0	0	0.785
DCA13	59	0	0	0	0	0.735
Mean				0.0169	0.0085	0.854
Total	590	10	10			

**FIGURE 2:**
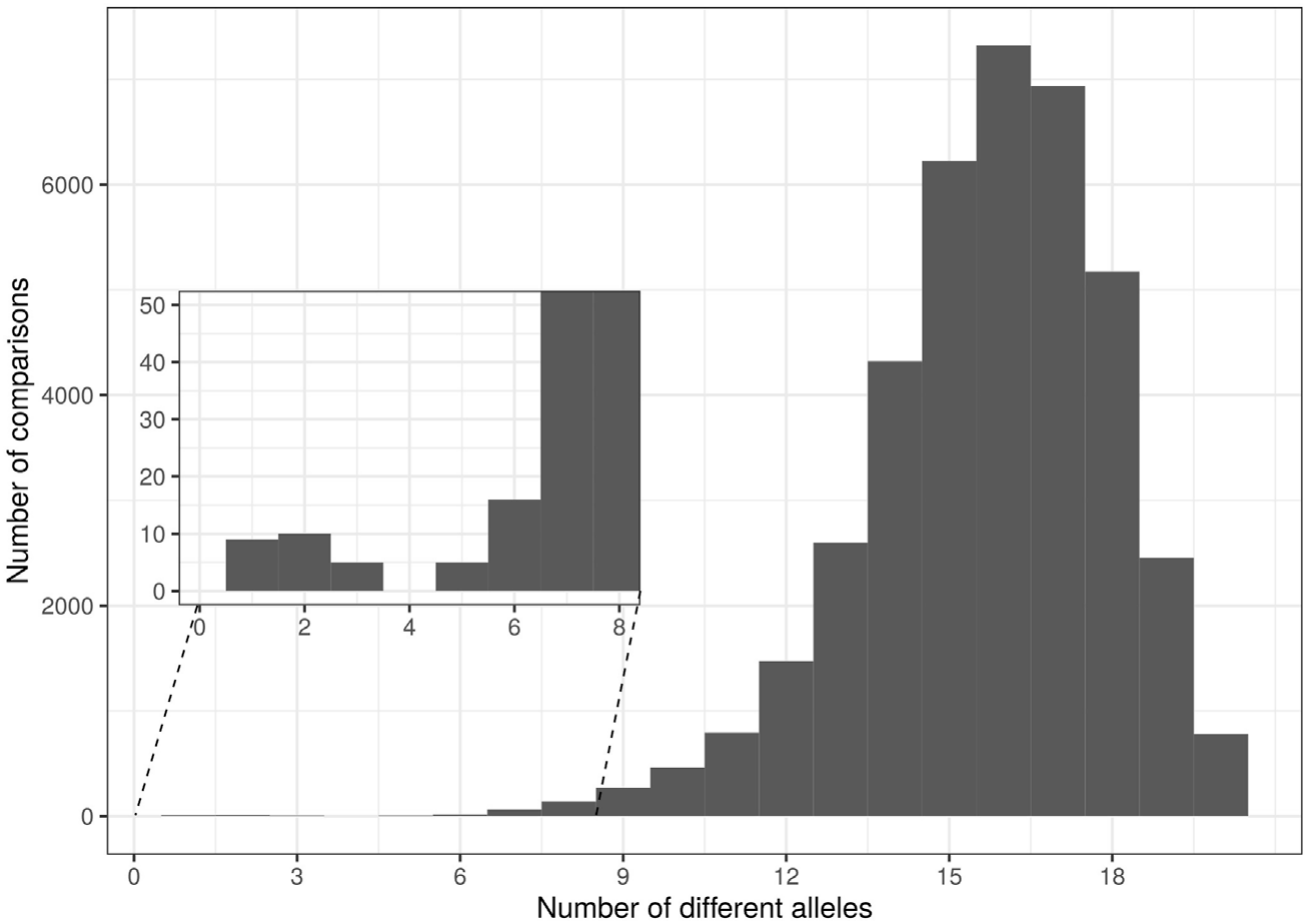
Histogram of pairwise genetic distances among all investigated olive tree samples.

Five cases of synonymy-clonality were detected (Table 4), all of which concerned cultivar samples and two of those concerned the NOGB. The latter were the sample pairs “Koutsourelia” - “Rachati”, and “Pierias (Skotinis)” - “Pierias”. The rest concerned cultivar samples from Ikaria which were identified as “Koroneiki” cultivar and one cultivar sample from Lesvos which was identified as “Chondrolia Chalkidikis”. Two cultivar samples from Crete had identical multilocus genotype. Additionally, a histogram of genetic distances between samples (each unit of distance equals one allele of difference) displayed a small peak at 1-3 distance units (Figure 2). The individuals forming this peak had 17–19 common alleles out of 20; hence they were regarded as “contracted MLGs” following the “contracted” multilocus genotype analysis available in poppr. These six contracted MLGs mostly involved cultivars as well. The reference sample “Chrysolia” had a match with one outgroup and reference samples “Chondrolia Chalkidikis” and “Chalkidikis” matched at 19/20 alleles. Two samples from olive groves in Lesvos were identified as “Valanolia” and one more sample from Lesvos was matched to “Adramytini” reference. Based on the overall results, the three samples from olive groves in Crete were all matched to “Mastoidis/Tsounati”. Finally one putative wild sample from Central Greece, two cultivar samples from Ikaria, reference sample “Throubolia”, commercially available “Throuba” and the monumental tree “Throuba Naxou” formed the last contracted MLG. The putative wild sample was removed from all further analyses. Only one sample from each MLG was included in analyses of genetic structure to avoid bias. There remained three cases of samples from olive groves of Lesvos (Ls-C-N-01, Ls-C-N-04, Ls-C-N-07) for which it was not possible to match them to a cultivar of the national collection.

**TABLE 4:** Synonyms and contracted MLGs among wild and cultivated olive samples.

Synonyms						
1	Ik-C-N-02	Ik-C-N-04	Koroneiki			
2	Ls-C-N-02	CHONDROLIA CHALK				
3	PIERIAS (SKOTINIS)	PIERIAS				
4	KOUTSOURELIA	RACHATI				
5	CR-C-N-01	CR-C-N-02				
Contracted MLGs						
1	CHRYSOLIA	OUT-1				
2	CHONDROLIA CHALK	CHALKIDIKIS				
3	Ls-C-N-03	Ls-C-N-06	VALANOLIA			
4	Ls-C-N-05	ADRAMYTINI				
5	CR-C-N-02	CR-C-N-03	MASTOIDIS_TSOUNATI			
6	CG-W-R-02	Ik-C-N-01	Ik-C-N-03	THROUBOLIA	THROUBA	THROUBA NAXOU

### 3.2 Wild population and cultivar genetic diversity, inbreeding and genetic differentiation

For wild populations in the 10 loci studied, 211 alleles were observed. The number of alleles per locus ranged from 15 (DCA13) to 30 (DCA16), with a mean of 21.1. The effective number of alleles ranged from ne = 4.558 (DCA13) to ne = 14.535 (DCA09), with a mean of ne = 9.109. Observed heterozygosity ranged from 0.396 (DCA13) to 0.885 (IAS.oli23), and gene diversity (Nei, 1978) ranged from 0.780 (DCA13) to 0.931 (DCA09) (Supplementary Table S3).

**FIGURE 3:**
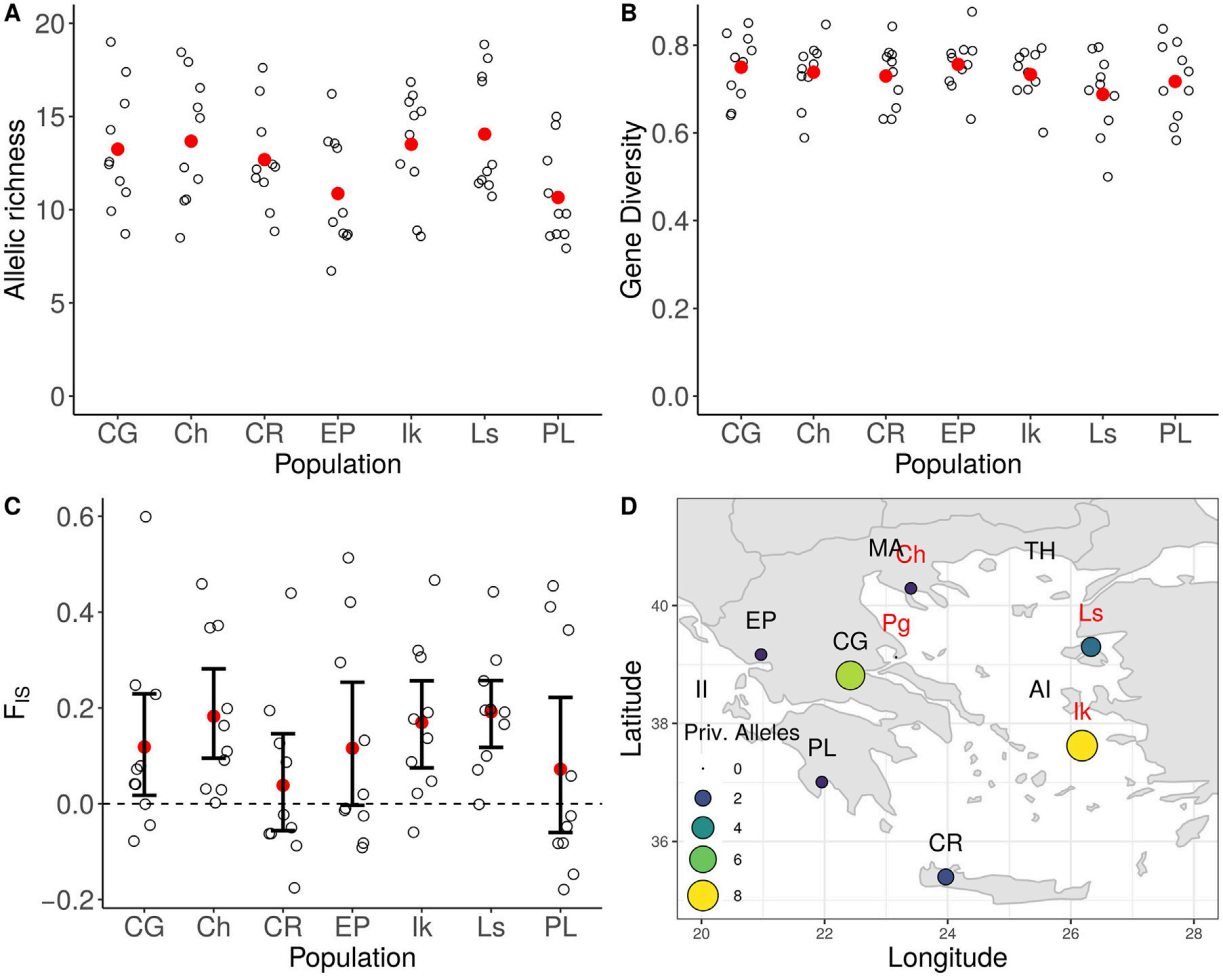
Genetic diversity statistics for wild olive tree populations. **(A)** Allelic richness, **(B)** Gene Diversity, **(C)** Inbreeding coefficient F_IS_, **(D)** Geographical distribution of private alleles.

All wild populations had mean allelic richness (AR) values > 10, with the lowest value observed in the population of the Peloponnese (AR = 10.65) and the highest in the population of Lesvos (AR = 14.05). Conversely, the population of Lesvos exhibited the lowest observed heterozygosity value (H_O_ = 0.695), while the highest was recorded in the population of Crete (H_O_ = 0.825). All loci presented positive values of the inbreeding coefficient FIS, except for the one corresponding to marker IAS. oli23 (−0.001). The mean F_IS_ value was 0.156. The 95% confidence intervals for the inbreeding coefficient F_IS_ were estimated to range above zero for the populations of Central Greece, Chalkidiki, Ikaria, and Lesvos. Twenty-three private alleles were detected among wild populations. Only the population of the Pagasitikos Bay did not carry any private allele, while eight were identified in the population of Ikaria (Figure 3).

Genetic distances among the wild populations ranged at low levels, with the lowest G_ST_ value detected between the populations of Lesvos and Central Greece (G_ST_ = 0.001, *p* = 0.220) and the highest between Lesvos and Peloponnese (G_ST_ = 0.032, *p* < 0.001). Due to the expected behaviour of G_ST_ to present low values when calculated from hyper-variable markers such as microsatellites, its standardized version G’‘_ST_ (Meirmans and Hedrick, 2011) and Jost’s D (Jost, 2008) are also available in Supplementary Figure S1.

Cultivated varieties displayed 127 alleles and the number of alleles per locus ranged from seven (DCA13, DCA14) up to 20 (UDO43). The lowest value for effective number of alleles ne = 2.175 was observed for marker DCA05 and the highest ne = 10.520 for DCA09, while the mean value was ne = 6.313. Mean observed heterozygosity Ho = 0.787 was slightly higher than the overall value detected in wild populations. Conversely, gene diversity uHe = 0.806 was lower than the corresponding value in wild olive trees (Supplementary Table S4).

### 3.3 Genetic differentiation between wild and cultivated olive trees

**FIGURE 4:**
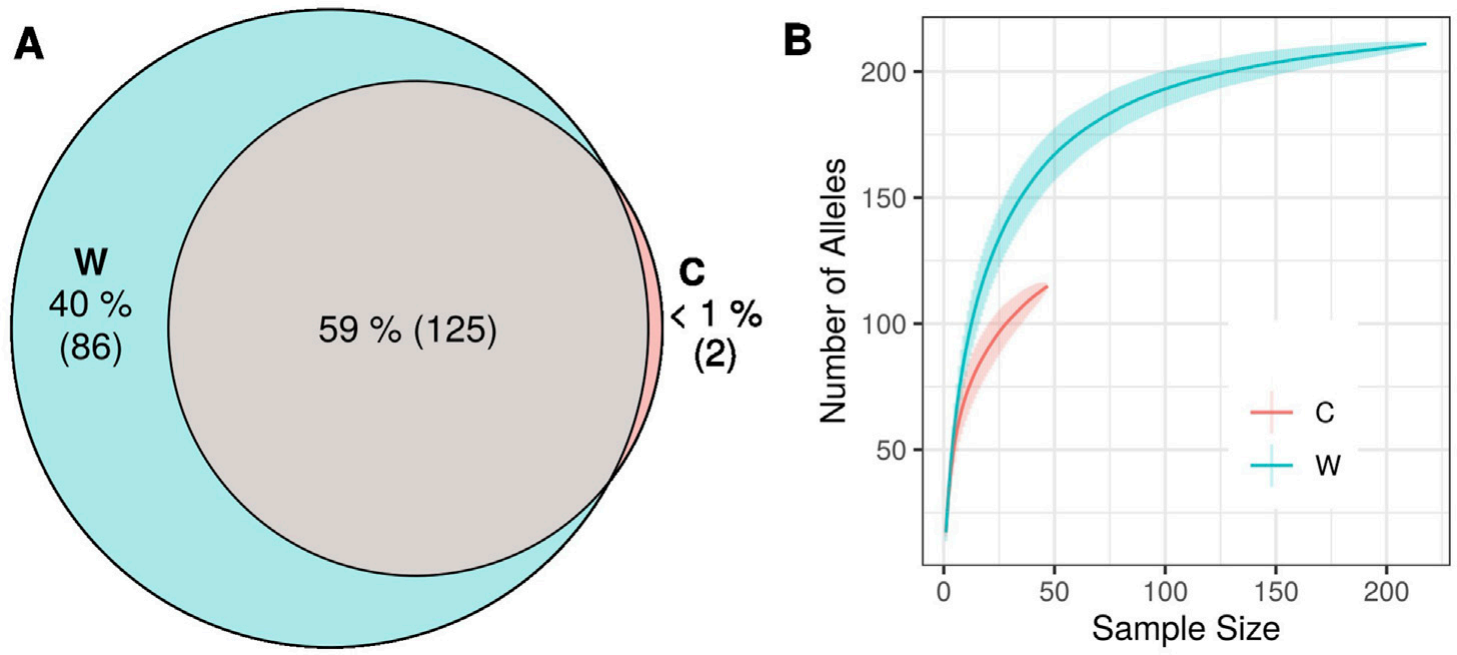
**(A)** Venn diagram of total number of alleles detected in the data set partitioned between wild and cultivated olive tree samples. **(B)** Rarefaction curves of number of alleles in wild and cultivated samples.

Two alleles (<1% of all alleles) were exclusively found in cultivars, while 86 alleles (40% of the total alleles) were only present in wild populations (Figure 4A). Rarefaction curves showed a substantially higher number of alleles for wilds compared to cultivars, given similar sample sizes (Figure 4B). The genetic differentiation between wild genotypes and Greek cultivars, was low (G_ST_ = 0.014) but statistically significant (*p* < 0.001). The complementary differentiation measures G’’_ST_ = 0.175, *p* < 0.001 and D = 0.151, *p* < 0.001 were also recorded.

**FIGURE 5:**
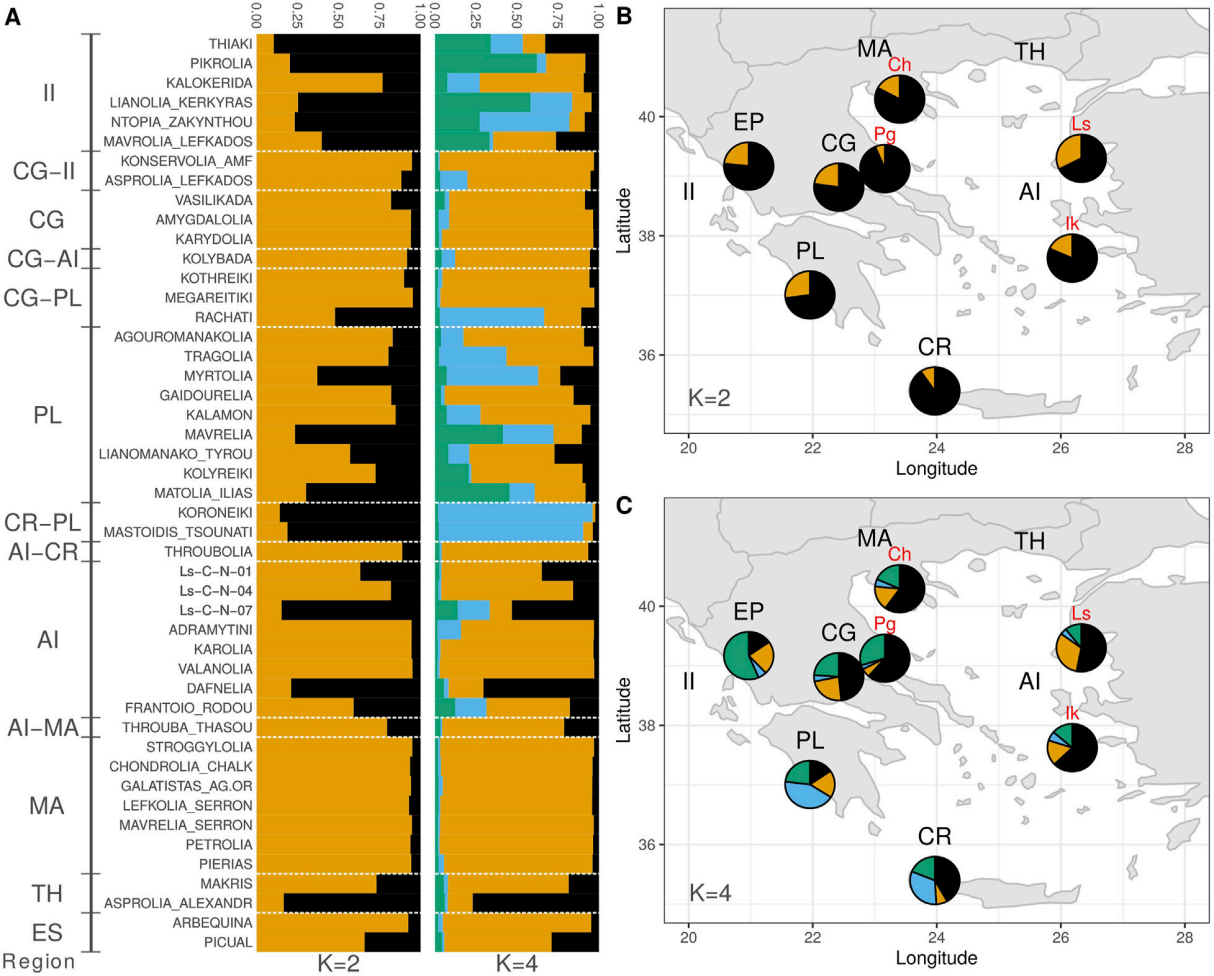
**(A)** Bayesian clustering of cultivated olive tree samples for K = 2 and K = 4. **(B)** Bayesian clustering of wild olive tree samples for K = 2. **(C)** Bayesian clustering of wild olive tree samples for K = 4.

For a better understanding of the genetic structure, Bayesian clustering was employed via the STRUCTURE software. Because locus DCA13 exhibited Hardy-Weinberg disequilibrium in six out of the seven populations tested, the analysis was performed with and without the presence of this locus. Due to the similarity of the results of both analyses, data obtained from all available loci is presented. Τhe optimal value of K groups was decided based on plotting the average of the estimated natural logarithm of the probability of the data indicating values of K = 3 and K = 4. Τhe Evanno method (Evanno et al., 2005) indicated a value of K = 2 (Supplementary Figure S3). For K = 2, the analysis indicated one group in which the majority of the cultivars were classified and another group containing the largest subset of wild genotypes (Figure 5). For K = 4, results demonstrated geographic coherence. A new group appeared, which was consistent with the geographical area of ​​Southwestern Greece (light-blue color), with the main representatives being the cultivars "Koroneiki” and "Mastoidis/Tsounati”, and high membership coefficients in the cultivars "Ntopia Zakynthou”, "Myrtolia”, "Rachati” and "Tragolia”. In wild genotypes, the largest subset of samples in the Peloponnese and Crete participated in the same group. Furthermore, another group was formed, which was mainly associated with the geographic area of Western Greece (green color). Specifically, high membership percentage was observed in two cultivars of the Ionian Islands (“Pikrolia”, "Lianolia Kerkyras”) and lower percentages in the cultivars "Thiaki”, "Mavrelia” and "Matolia Ilias”. Almost all samples of the wild population of Epirus and some samples from other geographic regions showed high similarity to this group. Most of the cultivars from the regions of Macedonia, Central Greece, the Aegean Islands and Thrace, as well as some cultivars of the Ionian Islands and Peloponnese, made up the most populous group of cultivars (orange color). It is worth noting that Spanish cultivars "Arbequina” and "Picual” had high membership proportions for the same group, similarly to some wild genotypes from Lesvos, Central Greece, Chalkidiki and Ikaria. Finally, the cultivars "Asprolia Alexandroupolis” and "Dafnelia” showed high participation rates in the last group of genotypes (black color), which included a large number of wild samples from the regions of Lesvos, Ikaria, Chalkidiki, Central Greece, and Pagasitikos Bay, forming a cluster that corresponds to the geographic area of the Aegean Sea.

**FIGURE 6:**
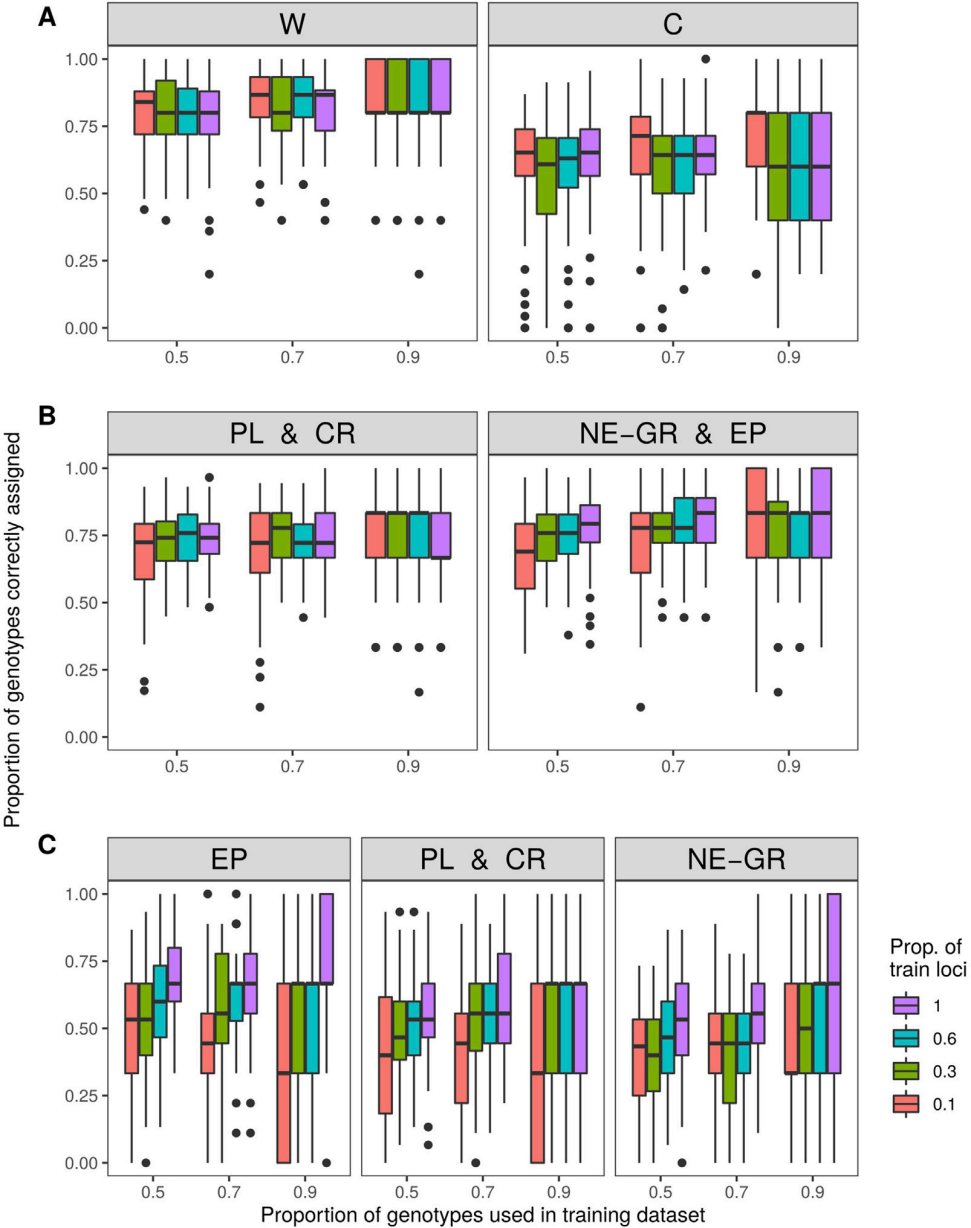
Assignment accuracy after 100 iterations with three levels of training samples (50%, 70%, 90% of samples) and four levels of training loci (1, 3, 6, 10 loci). Investigated groups: **(A)** wild vs. cultivated olive tree samples, **(B)** within wild olive tree samples from Peloponnese and Crete vs. North-Eastern Greece and Epirus, and **(C)** within wild olive tree samples from Epirus vs. Peloponnese and Crete vs. North-Eastern Greece.

The subsequent assignment analysis was conducted with the following predefined groups based on STRUCTURE results: i) wild samples or cultivars, ii) Peloponnese and Crete wilds or North-Eastern Greece and Epirus wilds, and iii) Peloponnese and Crete wilds or Epirus wilds or North-Eastern Greece wilds. To avoid bias related to unequal sample sizes, care was taken to use a balanced “training” data set (e.g., for the first comparison, 45 cultivars and 48 wild samples - six from each population - were used). On average 83% of wild genotypes and 68% of cultivars were assigned to the correct gene pool (“wild” or “cultivars”) (Figure 6A). When only wild individuals were taken into account and were separated into two groups "Peloponnese and Crete” (PL and CR) and "Northern-Eastern Greece and Epirus” (NE-GR) according to the STRUCTURE analysis results for K = 3, on average 69% of genotypes with Peloponnese or Crete origin and 69% of the remaining wild individuals were assigned to the correct group. These percentages increased to 73% and 71% respectively, when three random loci were used (Figure 6B). In the case of separating wild samples into three groups, 61% of individuals were correctly assigned to the population of Epirus (EP), 64% were correctly assigned to the group formed by the populations of Peloponnese and Crete (PL and CR), and 57% to the group of Northern-Eastern Greece (NE-GR) (Figure 6C).

### 3.4 Gene flow

To investigate the demographic history of the identified gene pools, a maximum likelihood tree was constructed using the TreeMix software. Similarly to the assignment analysis, populations were grouped based on the Bayesian clustering output. The wild olive genotypes were classified into three groups: "Northeastern Greece” (NE-GR), "Peloponnese-Crete” (PL and CR), and "Epirus” (EP). Most cultivars were included in a separate group (C1 - orange color in the STRUCTURE analysis), while the remaining cultivars, which had a membership percentage of <50% in group C1, were included in a second group (C2) since they mostly constituted "mosaics” of the three groups wild samples were members of (Supplementary Table S5).

**FIGURE 7:**
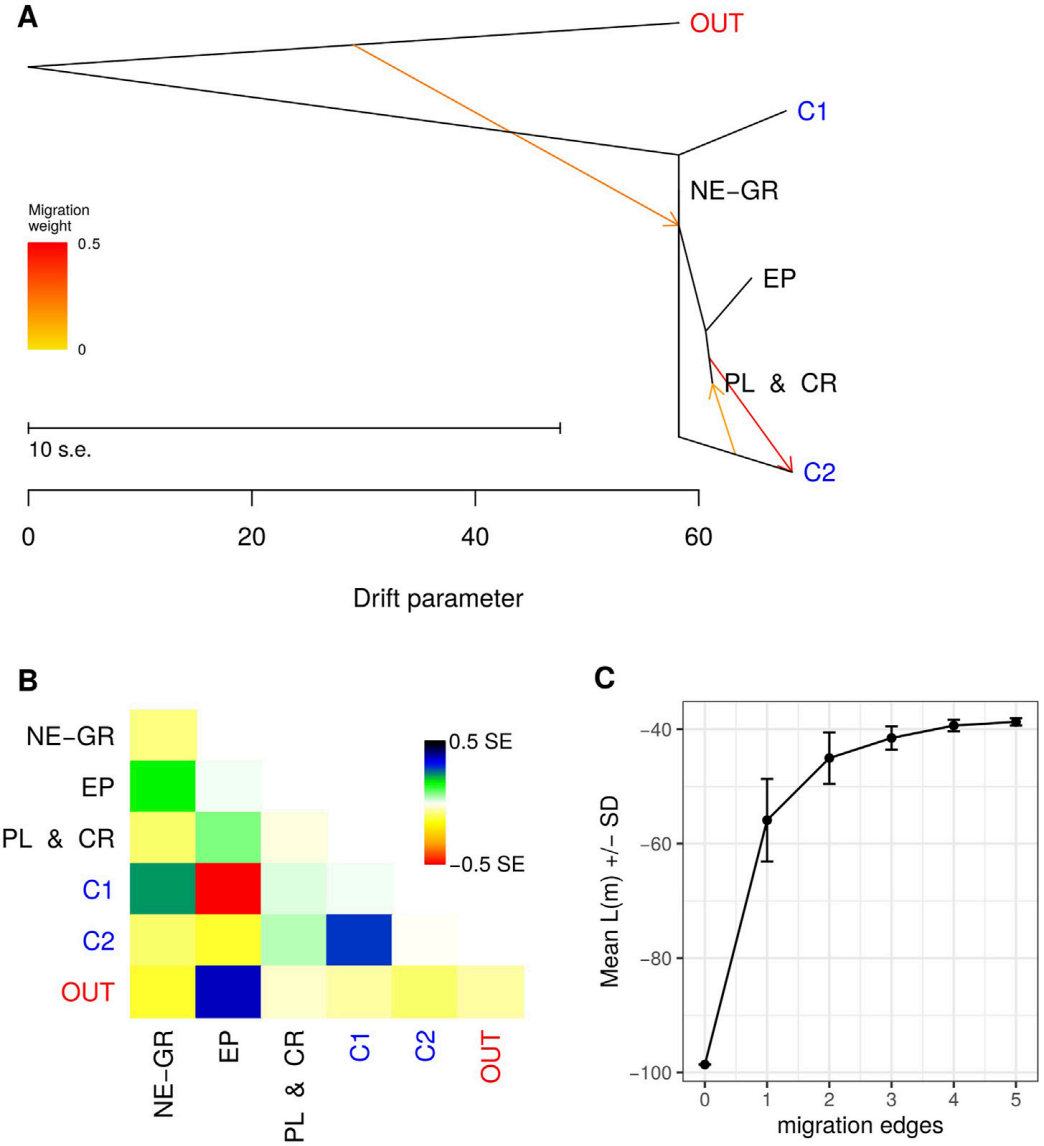
**(A)** Maximum Likelihood dendrogram of wild and cultivated olive tree sample groups as indicated by Bayesian clustering with three gene flow events inferred by TreeMix. **(B)** Residuals of the TreeMix model. **(C)** Mean likelihood values for none up to five gene flow events.

Based on the progression of likelihood values for different numbers of migration events (Figure 7C), the three-event model was selected. Τhe 10 independent repetitions of the model did not yield identical results, nevertheless the dendrogram presented in Figure 7Α resulted from 5 repetitions and attributed the relationships among the gene pools with the lowest standard errors compared to the remaining iterations. The first migration event indicates that 24% of the origin of the populations in Northeastern Greece (NE-GR) originates from a parental population that resembled genetically *O. europaea* subsp. *Cuspidata*. According to the second detected gene flow event, the C2 cultivar group emerged from gene transfer (migration intensity w = 48%) from populations in Peloponnese and Crete (PL and CR). Finally, a third incident of gene flow with the opposite direction compared to the second event, but with distinctly lower intensity (w = 15%).

## 4 Discussion

### 4.1 Quality check and statistical power

Error rates per reaction (average 1.69%) and per locus (average 0.85%), were at sufficiently low levels, ensuring the reproducibility of molecular analyses. In a similar study aimed at compiling a list of recommended loci for genotyping olive cultivars, Baldoni et al. (2009) selected 11 SSRs from a set of 37, after conducting molecular analysis that was independently repeated in four laboratories. For the 11 suggested loci (eight of which were also used in this study), the error rate per locus was calculated to be 2.26%. As for the frequency of errors among loci, it was observed that the most informative markers according to the PIC index, also had higher error rates. This phenomenon is previously reported (Flores-Rentería and Krohn, 2013) and has been observed in other species such as grapevine (Cipriani et al., 2008). One proposal for minimizing genotyping errors is to avoid highly polymorphic dinucleotide markers as they often come with issues related to false peaks, in favour of microsatellites with repeat motifs of three to five nucleotides (Dewoody et al., 2006).

The PID (1.60 × 10^−16^) and PIDsibs (1.25 × 10^−5^) indices showed low values, confirming the expected high discriminatory power to identify cases of clonality within the dataset. Compared to other studies, Haouane et al. (2011) genotyped the worldwide olive genetic bank of Marrakech, Morocco (OWGB) and recorded an overall PID of 2.55 × 10^−14^. This value was calculated using 12 microsatellite loci and 210 associated alleles. In another application involving 431 samples from wild and cultivated trees in the Spanish region of Andalusia, with 14 SSR loci and 191 alleles, a value of PID = 1.96 × 10^−16^ was estimated (Díez et al., 2011), which is in the same order of magnitude as this study. Although Haouane et al. (2011) detected more alleles than Diez et al. (2015), the identity index was higher in the latter case. This is probably due to the fact that allelic frequencies between wild and cultivated gene pools differ enough, so that fewer alleles are required to distinguish samples with the same level of confidence. In addition, the discriminatory power of genetic markers is likely to be lower for cultivar samples which bear to some extent the imprint of genetic bottlenecks as a result of the domestication process (Diez et al., 2015).

### 4.2 Clone detection

Eleven out of 14 samples from local olive groves in Crete, Lesvos, and Ikaria, which were adjacent to the sampling areas of wild genotypes, were successfully matched to known cultivars. Regarding the reference samples, the cultivar pairs "Pierias” - "Pierias (Skotinis)", "Koutsourelia” - "Rachati”, and "Chondrolia Chalkidikis” - "Chalkidikis”, displayed identical, or highly similar multilocus genotypes, and were treated as clones. However, they possess morphological differences (Koubouris et al., 2019) and it is hypothesized that one member of each pair originated from undetected mutations from the other. Similar cases have also been suggested for cultivar pairs maintained at the World Olive Germplasm Bank of Cordoba (Trujillo et al., 2014). It is worth mentioning, that in the recent work by Bazakos et al. (2023) employing whole genome re-sequencing, the "Chondrolia Chalkidikis” - "Chalkidikis” pair had a proportion of genomes shared identical-by-descent PropIBD = 0.9819, while a PropIBD = 0.8801 was recorded for "Koutsourelia” - "Rachati".

Additionally, the "Chrysolia” accession was found to share the same contracted MLG with one of the outgroup samples and therefore should be classified as *O. europaea* subsp. *Cuspidata*. The reference sample of the national collection "Throubolia,” the commercially available sample "Throuba” and the over 3,000 years old monumental olive tree "Throuba Naxou” formed another contracted MLG (also confirmed by Bazakos et al., 2023). “Throubolia” cultivar possesses the unique agronomic characteristic of fruit ripening and being instantly edible after harvesting (Kostelenos and Kiritsakis, 2017). Such a trait is expected to have been highly desirable during the domestication process and the identification of this monumental tree supports the hypothesis that the Greek geographic region served as an ancient hotspot for olive cultivar development. This hypothesis could hold true even if the Greek geographic region at large, did not act as a primary centre of olive domestication.

### 4.3 Genetic diversity within wild gene pool

All populations of wild olive trees showed high levels of genetic diversity, as evidenced by the high values of allelic richness and gene diversity, a result which is expected for a species that is characterized by a complex self-incompatibility system (Koubouris et al., 2014; Breton et al., 2021). In all markers but IAS. oli23, a heterozygote deficiency was observed, which might be linked to small population sizes, limited gene flow between fragmented local populations and/or genetic bottlenecks.

The populations of Epirus and of the Peloponnese displayed slightly lower allelic richness values than the rest. In the populations of Ikaria and Central Greece, eight and seven private alleles were found respectively, while in the rest of the populations, one to three private alleles were recorded (excluding Pagasitikos Bay). This pattern may reflect the intensity of genetic pollution, with areas of historically lower oleiculture intensity maintaining a higher percentage of rare alleles. Undoubtedly, the search for wild individuals, whether true-to-type wild olives or ferals, should focus on geographical areas far from places with a long tradition of olive cultivation.

### 4.4 Genetic differentiation between wild and cultivated olive trees

The level of genetic differentiation between wild and cultivated olive gene pools, as expressed by the G_ST_ coefficient, was low although statistically significant (G_ST_ = 0.014, *p* < 0.001). Low levels of genetic differentiation between the two pools have also been proposed in most regions of the Mediterranean (Belaj et al., 2007; Hannachi et al., 2008; Diez et al., 2015). The absence of strong differentiation signals indicates that the collected wild olive samples should probably not be considered "true-to-type” wilds, but rather feral forms (i.e., individuals that have arisen from multiple admixture events between wild ancestors and cultivated genotypes). However, it should be noted that levels of genetic differentiation observed in the olive tree are lower compared to other perennial tree species, partly due to the long time between successive generations (Besnard et al., 2018). A proper characterization as wild or feral, would require reference samples of wild genotypes and employment of more phylogenetically informative marker systems, such as organelle microsatellites or high throughput sequencing.

Almost all (>99%) of cultivar alleles were detected in wild individuals. In contrast, 40% of the total alleles were recorded only in the wild populations. This difference in the number of private alleles was not a statistical artifact due to differential sampling effort, as confirmed by rarefaction curves which demonstrate that for similar sample sizes the cultivated gene pool withholds fewer alleles. Rarefaction curves provide valuable conclusions when samples are representative of each group. The stratified sampling over eight broad geographical areas for wild genotypes, ensures that this condition is met. Ensuring the same for cultivars, i.e., that accessions which make up the national collection constitute an accurate representation of the total genetic diversity that is found in the country’s cultivated plant genetic material, is more challenging. However, it can be considered as a reasonable assumption since the origin of cultivars that are included in NOGB spatially covers all olive cultivation regions in Greece. This is the first study that reports the extent of the wealth of unique genetic variation present in wild olive gene pools in Greece. The finding indicates that wild samples cannot have emerged exclusively from cultivars escaping cultivation. It also corresponds to a genetic bottleneck as a result of domestication as previously recorded by Besnard et al. (2013b) who reported a loss of genetic diversity of around 16% (for nuclear loci) and 64% (for chloroplast loci) in cultivated olive genotypes compared to wild individuals. Similar results have also been reported by Lumaret et al. (2004) where significantly lower heterozygosity was found in cultivars compared to wilds from the eastern and western Mediterranean.

The Bayesian analysis performed using the STRUCTURE software clustered together several cultivars with the same origin/principal area of cultivation, especially in the Peloponnese, Crete, and Epirus regions. Cultivars were not strictly clustered according to region though. Therefore, the evolution of olive cultivated germplasm in Greece may have been influenced by human-mediated gene flow of improved clones, as has been hypothesized by prior studies (Nikoloudakis et al., 2003; Owen et al., 2005; Roubos et al., 2010). Additionally, two cases of genetic similarity were noted concerning cultivars from Ionian islands with Epirus wilds, and cultivars from Peloponnese-Crete with wild samples from the same regions. For the Chania (Crete) region where wild sampling for the present study was conducted, Aksehirli-Pakyurek et al. (2017) identified genetic relatedness between wild samples and the "Mastoidis” cultivar. Taken together, these findings point to genetic pollution from historical human agricultural activity which has most likely eroded the wild gene pool. However, evidence from organelle DNA revealed that the Peloponnese area could be a contact spot between the two known wild olive gene pools of West and East Mediterranean (Besnard et al., 2013a; 2013b). Hence, the spatial structure that wild populations exhibited could also be attributed up to some extent to a phylogeographic signal.

Although a signal of genetic structure was detected among the wild olive populations, the rates of correct assignment were low. This means that assigning samples of unknown origin to geographical regions is not expected to have high reliability. Rates of correct assignment are expected to improve with an increase in sample size. Benestan et al. (2015, 2016) recorded 10,156 SNPs in lobster populations and showed that the success of assignment was substantially affected by the intensity of sampling. Specifically, for sample sizes <50, the success of assignment was limited to approximately 60%, slightly higher than the 50% expected if assignment was randomly performed. Conversely, for population sizes ≥100, successful assignment rates higher than 80% were recorded. Although collecting a large number of individuals (≥100) seems to offer high reliability in genotype assignment analyses, increasing the sample size is not always feasible, particularly in populations that have undergone bottlenecks and/or genetic pollution, as is the case for wild olive trees. Assignment rates could also be improved without increasing the sampling effort in the number of samples or genetic markers, by including morphological data. Rellstab et al. (2016) characterized almost 1,400 samples of phylogenetically close oak species in Switzerland using eight microsatellite markers and 13 morphological traits. The highest successful assignment rate was achieved by combining molecular and morphological data.

### 4.5 Gene flow

By investigating the data set using the software TreeMix, three migration events emerged. The first one indicated gene flow from an ancestral population of *O. europaea* subsp. *Cuspidata* to the populations of north-eastern Greece (NE-GR). Hybridization with other subspecies has been found in multiple studies, for example, the Dhokar cultivar shares genetic origins with subspecies *laperrinei* (Besnard et al., 2013b). Genetic affinity between populations of *O. europaea* subsp. *Cuspidata* in Egypt and wild olive *O. europaea* var. *sylvestris* in the Eastern Mediterranean has also been reported (Besnard et al., 2007). However, the exchange of genetic material between the two subspecies appears to be limited to ancestral hybridization events before the creation of the natural barrier of the Sahara desert (Besnard et al., 2012; 2013b). Thus, the observed genetic link might represent an ancient event which took place in the Eastern - South-eastern Mediterranean or more likely a recent event connected to cultivar dispersion beyond the Mediterranean (Besnard et al., 2018).

The next two events involved bidirectional gene flow between the population of wild olives in Peloponnese-Crete (PL and CR) and the cultivar group C2, which was distinguished based on the clustering results and contained almost exclusively cultivars from Peloponnese, Crete and Epirus. Gene flow intensity was higher in the direction from wild populations to cultivated individuals, which possibly reveals the existence of secondary domestication events of limited geographical scope in Greece. Nevertheless, the bidirectional aspect of this gene exchange between the two groups also indicates wild population genetic erosion.

### 4.6 Implications for olive tree genetic resources management in Greece

The present study demonstrates that Greek wild olive tree populations withhold high levels of genetic diversity, a large proportion of which is not present in the cultivated germplasm. This is of paramount importance, regardless of the degree to which current wild individuals resemble their wild ancestors and contemporary cultivars. Although the presence of high genetic diversity in wild populations indicates that they are not at risk of immediate collapse, caution should be exerted to the fact that their ecological habitat is fragmented and surrounded by olive groves. Therefore, unless conservation measures are taken, the genetic diversity and identity of future wild olive tree generations are expected to be eroded to a higher extent than what has already occurred. The establishment of *in situ* protected areas is proposed for the conservation of isolated populations. Candidate areas have been proposed in Martsalo Gorge (Asterousia Mountains, Crete) and Mount Mandalo south of Rethymno (Banilas et al., 2009). It is expected that in such areas wild populations will be able to continue adapting to future environmental changes. For the vast majority of Greek wild populations though, geographical isolation from olive groves is not realistic. Hence the establishment of *ex situ* conservation units away from olive grove pollen flow is the only solution to maintain their genetic integrity in the long term. The selection of individuals for such a collection could be aided by the use of molecular tools, such as those used in this study, to collect plant genetic material from all genetic groups found in the country. A special case is that of centennial trees found in traditional olive groves. Many of them were propagated through grafting onto wild rootstocks, which means that they retain at their base a snapshot of the genetic diversity that existed in each region hundreds of years ago. Recording and protection of these olive groves is of the utmost importance. Finally, in a rather limited set of 14 cultivar samples from olive groves, three of them (all from Lesvos island) were not matched to any of the reference samples from the Greek National Germplasm Bank. Growers characterized these unidentified trees as local cultivars, which underscores the need for thorough recording and conservation of the country’s cultivated germplasm.

### 4.7 Utilization of wild olive trees in breeding, use as rootstocks

The domestication process from which olive cultivars have emerged is associated with genetic bottlenecks due to selection from local gene pools (Besnard et al., 2013a; Diez et al., 2015). Evidence from the present study as well as from prior works (please add references) suggest that wild olive tree populations exhibit unique genetic polymorphisms that are not found in the cultivated genetic material (Barazani et al., 2014; 2016; Mariotti et al., 2020; Zunino et al., 2023). Therefore, they are expected to constitute an invaluable source of variation for agronomic traits of interest. With regard to olive oil quality, Leon et al. (2018) studied the chemical composition of olive oil of cultivars, wild individuals and their hybrids. They concluded that the utilization of wild material did not have negative effects on fatty acid composition, tocopherol content, and tocopherol and phytosterol profiles if selection for these traits was carried out in the early generations of the breeding programs. In fact, it was found that tocopherol content could be improved by using wild parents. In another wild olive progeny test, higher vigour, a shorter juvenile phase and denser flowering were recorded compared to open pollination families of the cultivar “Picual” (Klepo et al., 2013). Interestingly, high resistance of wild olive genotypes to verticillium has been recorded, which in some cases was higher compared to the resistant cultivars (Colella et al., 2008; León et al., 2014). Wild olive trees are also regularly used as rootstocks with high resistance to biotic/abiotic factors as explained above. Research in rootstock management is also a very promising field of study, as there is evidence of non-random clustering of rootstocks and cultivars (Barazani et al., 2017). Therefore, the identification of unexploited adaptive traits in wild olive genetic resources and their potential utilization as rootstocks for olive cultivation, notably in Mediterranean-climate regions, could represent a major goal of olive crop breeding.

### 4.8 Limitations

Here we demonstrate that the 10 nuclear microsatellites analysed in this study were highly informative in discriminating olive tree accessions and elucidating patterns of genetic structure and gene flow. Nevertheless, using a high number of genomic markers in samples of a greater geographical area in the future would increase the robustness of such a study. In fact, a relatively low genetic differentiation was recorded between wild and cultivated trees, as well as among wild populations. The precision of this rather weak signal of fine genetic structure might have been lessened by the employment of a limited number of markers (Wang, 2017). Genotyping of organelle genetic markers and especially the use of high throughput genome sequencing methods are expected to provide higher resolution to estimate trends of effective population sizes (Julca et al., 2020), accurately determine hybridization events among wild and cultivated olive tree gene pools (Bazakos et al., 2023), and elucidate olive tree evolutionary history (Julca et al., 2023). Moreover, as inferences regarding gene flow/minor domestication events are based on wild olive/cultivar reference samples that mostly originated within Greece, they should ideally be further validated with an extended data set.

## 5 Conclusion

The contemporary wild olive gene pool is unique, rich and unexplored. It is the result of the interaction of complex natural phylogenetic processes and anthropogenic influence through the indirect reinforcement of hybridization by the spread of improved germplasm. In the present study, molecular genetic analysis uncovered a total of 86 alleles (40% of the total number of alleles detected) which were not present in the Greek-origin cultivars. This fact demonstrates the existence of a diverse genetic resource which cannot possibly have originated purely from hybridisations between cultivars and therefore needs to be protected both *in situ* and *ex situ*. Special attention should also be given to centennial olive trees residing within traditional olive groves. As grafting with wild rootstocks was a highly common practice for grove establishment in Greece, these trees act as preservation vessels for potentially lost wild populations and should be explored and protected. From the perspective of cultivar breeding, the high allelic richness recorded is indicative of the potential of wild genetic material to be used in cultivar improvement and rootstocks. Genetic differentiation between wild individuals and cultivars was low and only weak genetic structure was observed between the wild populations. In addition, results from bayesian and gene flow analysis revealed signs of genetic pollution for populations from western Greece. Taken together, wild samples analysed in this work are probably best described as feral, with western Greek populations having endured higher genetic erosion than the rest which might resemble closer the genetic composition of their wild progenitors. Finally, the genetic profile matching of “Throuba Naxou” with “Throubolia” cultivar along with the uncovering of a gene exchange event from wild to cultivated gene pool are indications which support the hypothesized role of the Greek region as a hotspot of oleiculture evolution in the form of secondary domestication events.

## Data Availability

The datasets generated during the current study are available in the Zenodo repository, https://doi.org/10.5281/zenodo.8104610. Analysis code and a fully reproducible computational environment is available in the same repository.
