# Supplementary material for: Wild and cultivated olive tree genetic diversity in Greece: a diverse resource in danger of erosion
Source: Front Genet. 2023 Dec 4;14:1298565. doi: 10.3389/fgene.2023.1298565 (PMC10725918; doi:10.3389/fgene.2023.1298565)
Supplement: Supplementary file 1 [file DataSheet1.docx]

Supplementary Material

## Supplementary Figures

**
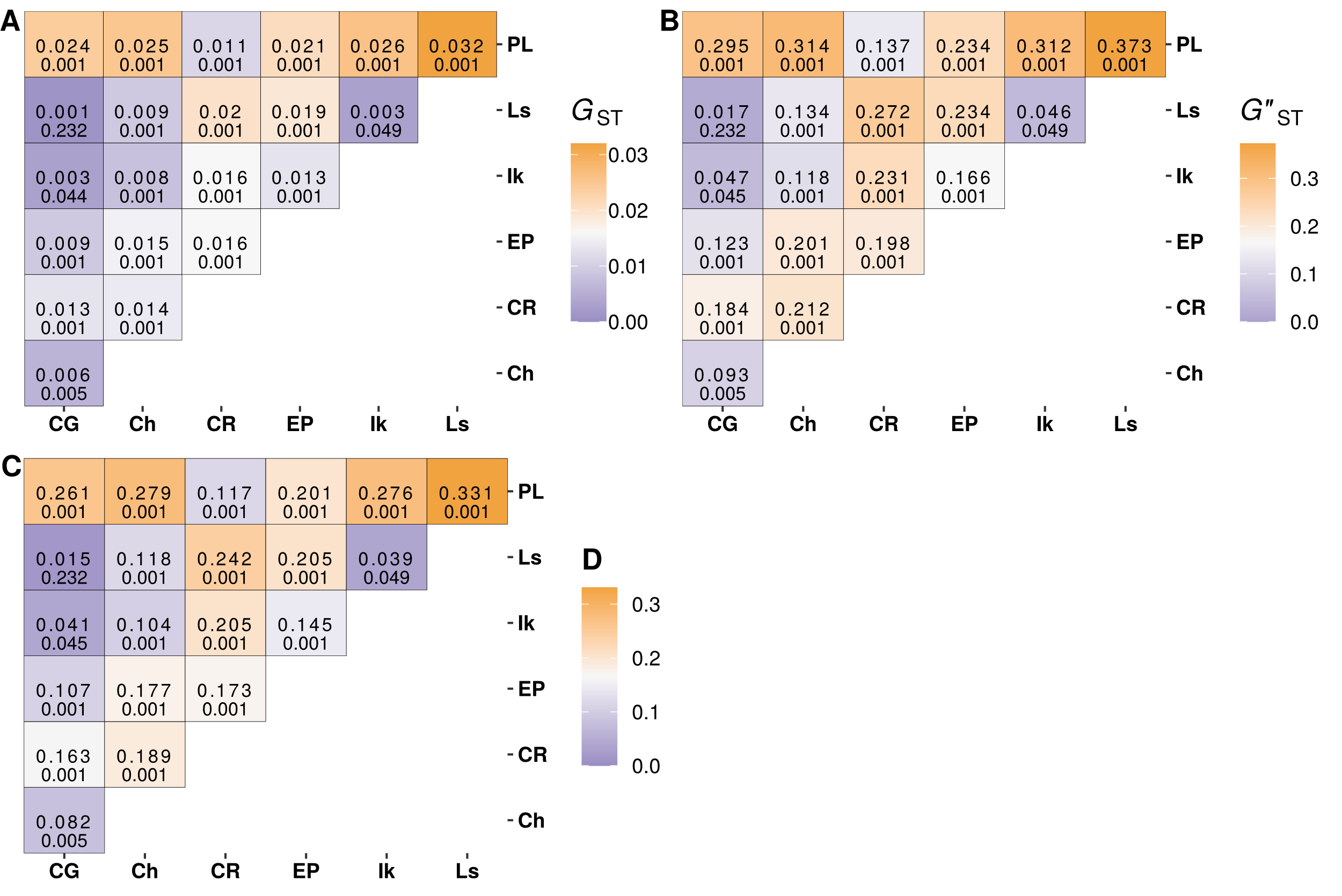
**

**Supplementary Figure S1.** Genetic differentiation measures between wild olive tree populations. Large font denotes effect size and small font denotes p-value. **(A)** G_ST_, **(B)** G''_ST_ (Meirmans and Hedrick, 2011), **(C)** Jost's D (Jost, 2008).


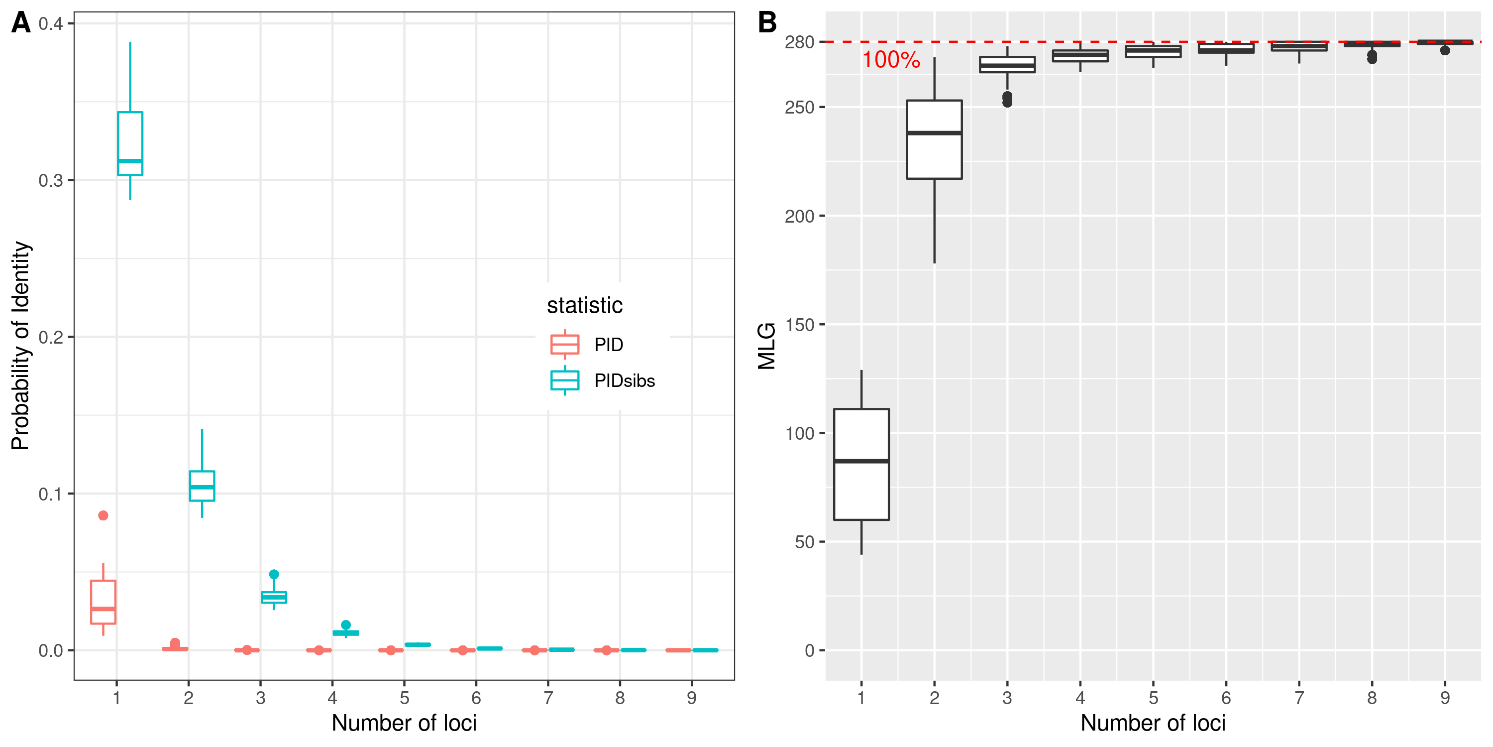


**Supplementary Figure S2.** **(A)** PID and PIDsibs median values from 1000 random sets of 1-9 genotyped loci., **(B)** MLG accumulation curve from 1000 random sets of 1-9 genotyped loci.


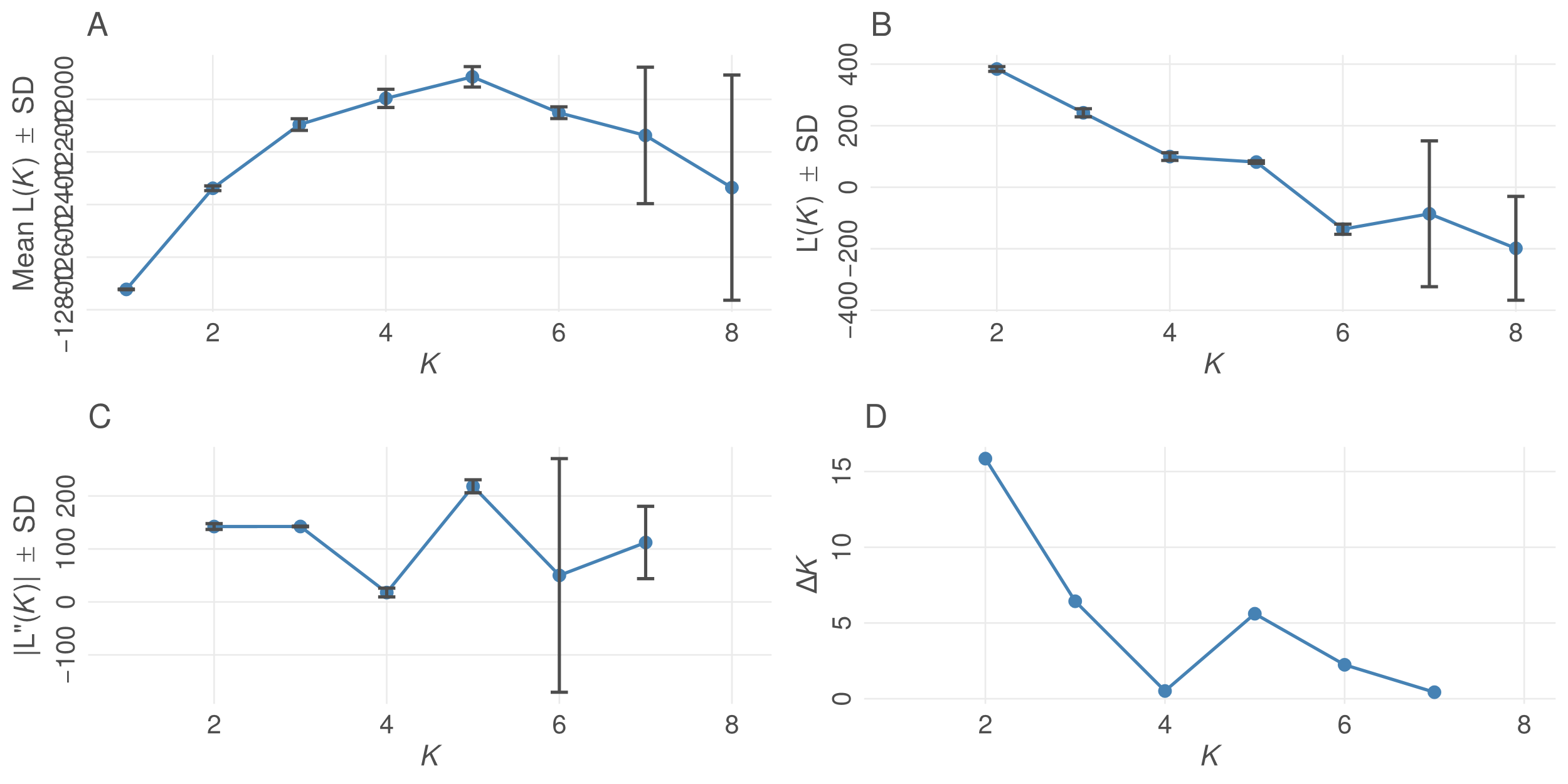


**Supplementary Figure S3.** Estimation of appropriate K number. **(A)** Average of the estimated ln of the probability of the data, **(B)** First derivative ln of the probability of data, **(C)** Second derivative ln of the probability of data, **(D)** Evanno's ΔK.

## Supplementary Tables

**Table S1.** Geographical coordinates of wild olive sampling plots

| **Population** | **Latitude** | **Longitude** |
| --- | --- | --- |
| Crete | 35,41374 | 23,95958 |
|  | 35,39528 | 23,96914 |
|  | 35,27255 | 23,72091 |
|  | 35,24985 | 23,69265 |
| Chalkidiki | 39,99370 | 23,89331 |
|  | 40,29276 | 23,40222 |
|  | 40,23336 | 24,30491 |
| Lesvos | 39,03490 | 26,60915 |
|  | 39,01791 | 26,58227 |
|  | 39,05843 | 26,53638 |
|  | 39,14804 | 26,50292 |
|  | 39,25623 | 26,40917 |
|  | 39,29970 | 26,32876 |
|  | 39,13122 | 26,42531 |
|  | 39,16294 | 26,51778 |
|  | 39,12008 | 26,48730 |
|  | 39,12334 | 26,39455 |
|  | 39,10179 | 26,36165 |
|  | 39,16125 | 26,28857 |
|  | 39,19754 | 26,16969 |
|  | 39,13436 | 26,11760 |
| Peloponnese | 37,64190 | 21,61012 |
|  | 37,00679 | 21,95514 |
|  | 37,12424 | 21,97009 |
|  | 37,04552 | 22,18029 |
| Epirus | 39,16912 | 20,96757 |
|  | 39,10523 | 21,07778 |
| Central Greece | 38,81648 | 22,42947 |
|  | 38,81530 | 22,41980 |
| Ikaria | 37,61057 | 26,19035 |
|  | 37,62415 | 26,18365 |
|  | 37,62634 | 26,17896 |
| Pagasitikos Bay | 39,09946 | 23,08396 |
|  | 39,12571 | 23,16252 |
|  | 39,12909 | 23,17973 |

**Table S2.** PID and PIDsibs median values from 1000 random sets of 1-9 genotyped loci. Overall values for the whole data set are presented in the last row.

| **Loci** | **PID** | **PIDsibs** |
| --- | --- | --- |
| 1 | 2.63E-02 | 3.12E-01 |
| 2 | 7.09E-04 | 1.04E-01 |
| 3 | 2.12E-05 | 3.38E-02 |
| 4 | 4.46E-07 | 1.09E-02 |
| 5 | 1.27E-08 | 3.55E-03 |
| 6 | 3.39E-10 | 1.15E-03 |
| 7 | 8.56E-12 | 3.78E-04 |
| 8 | 2.26E-13 | 1.20E-04 |
| 9 | 7.15E-15 | 3.95E-05 |
| 10 | 1.60E-16 | 1.25E-05 |

**Table S3.** Genetic diversity statistics for wild olive tree samples after clone removal

| **Marker** | **N** | **na** | **ne** | **I** | **Ho** | **uHe** | **Fis** |
| --- | --- | --- | --- | --- | --- | --- | --- |
| DCA03 | 217 | 20 | 6.361 | 2.208 | 0.820 | 0.843 | 0.027 |
| DCA05 | 217 | 17 | 6.131 | 2.168 | 0.747 | 0.837 | 0.108 |
| DCA09 | 213 | 23 | 14.535 | 2.820 | 0.859 | 0.931 | 0.077 |
| DCA13 | 217 | 15 | 4.558 | 1.872 | 0.396 | 0.780 | 0.492 |
| DCA14 | 215 | 22 | 10.953 | 2.644 | 0.740 | 0.908 | 0.186 |
| DCA16 | 206 | 30 | 11.299 | 2.794 | 0.752 | 0.911 | 0.174 |
| DCA18 | 218 | 22 | 10.225 | 2.541 | 0.817 | 0.902 | 0.095 |
| GAPU71B | 212 | 19 | 5.621 | 2.089 | 0.741 | 0.822 | 0.099 |
| IAS.oli23 | 217 | 16 | 8.636 | 2.322 | 0.885 | 0.884 | -0.001 |
| UDO43 | 214 | 27 | 12.771 | 2.863 | 0.640 | 0.921 | 0.305 |
| Mean | 214.6 | 21.1 | 9.109 | 2.432 | 0.740 | 0.874 | 0.156 |

N: number of samples, na: number of alleles, ne: number of effective alleles, I: Shannon’s index, Ho: observed heterozygosity, uHe: gene diversity (Nei 1978), Fis: inbreeding coefficient

**Table S4.** Genetic diversity statistics for cultivar samples after clone removal

| **Marker** | **N** | **na** | **ne** | **I** | **Ho** | **uHe** | **Fis** |
| --- | --- | --- | --- | --- | --- | --- | --- |
| DCA03 | 47 | 11 | 6.831 | 1.974 | 0.915 | 0.854 | -0.072 |
| DCA05 | 47 | 8 | 2.175 | 1.177 | 0.511 | 0.540 | 0.055 |
| DCA09 | 47 | 15 | 10.515 | 2.428 | 0.894 | 0.905 | 0.013 |
| DCA13 | 47 | 7 | 3.802 | 1.473 | 0.383 | 0.733 | 0.480 |
| DCA14 | 47 | 7 | 3.741 | 1.508 | 0.638 | 0.732 | 0.129 |
| DCA16 | 47 | 15 | 7.148 | 2.162 | 0.872 | 0.860 | -0.014 |
| DCA18 | 47 | 12 | 8.110 | 2.205 | 0.894 | 0.877 | -0.019 |
| GAPU71B | 47 | 10 | 6.481 | 1.934 | 0.894 | 0.846 | -0.057 |
| IAS.oli23 | 47 | 10 | 6.231 | 1.944 | 0.957 | 0.841 | -0.141 |
| UDO43 | 47 | 20 | 8.097 | 2.434 | 0.915 | 0.877 | -0.044 |
| Mean | 47 | 11.5 | 6.313 | 1.924 | 0.787 | 0.806 | 0.033 |

N: number of samples, na: number of alleles, ne: number of effective alleles, I: Shannon’s index, Ho: observed heterozygosity, uHe: gene diversity (Nei 1978), Fis: inbreeding coefficient

**Table S5.** Grouping of cultivar samples based on Bayesian clustering results

| Sample | Cultivar_group |
| --- | --- |
| Ls-C-N-01 | C1 |
| Ls-C-N-04 | C1 |
| ADRAMYTINI | C1 |
| STROGYLOLIA | C1 |
| KAROLIA | C1 |
| VALANOLIA | C1 |
| THROUBOLIA | C1 |
| AGOUROMANAKOLIA | C1 |
| TRAGOLIA | C1 |
| GAIDOURELIA | C1 |
| KONSERVOLIA_AMF | C1 |
| KALAMON | C1 |
| VASILIKADA | C1 |
| KOTHREIKI | C1 |
| KOLYBADA | C1 |
| AMYGDALOLIA | C1 |
| CHONDROLIA_CHALK | C1 |
| KALOKAIRIDA | C1 |
| KARYDOLIA | C1 |
| MEGARITIKI | C1 |
| FRANTOIO_RODOU | C1 |
| GALATISTAS_AG.OR | C1 |
| THROUMPA_THASOU | C1 |
| LEFKOLIA_SERRON | C1 |
| LIANOMANAKO_TYROU | C1 |
| KOLYREIKI | C1 |
| MAVRELIA_SERRON | C1 |
| PETROLIA | C1 |
| ASPROLIA_LEFKADOS | C1 |
| PIERIAS | C1 |
| MAKRIS | C1 |
| ARBEQUINA | C1 |
| PICUAL | C1 |
| Ls-C-N-07 | C2 |
| THIAKI | C2 |
| MYRTOLIA | C2 |
| DAFNELIA | C2 |
| PIKROLIA | C2 |
| MAVRELIA | C2 |
| LIANOLIA_KERKYRAS | C2 |
| KORONEIKI | C2 |
| RACHATI | C2 |
| MASTOIDIS_TSOUNATI | C2 |
| MATOLIA_ILIAS | C2 |
| NTOPIA_ZAKYNTHOU | C2 |
| MAVROLIA_LEFKADOS | C2 |
| ASPROLIA_ALEXANDR | C2 |
